# Positive Allosteric Modulator of GABA Lowers BOLD Responses in the Cingulate Cortex

**DOI:** 10.1371/journal.pone.0148737

**Published:** 2016-03-01

**Authors:** Susanna A. Walter, Mikael Forsgren, Karin Lundengård, Rozalyn Simon, Maritha Torkildsen Nilsson, Birgitta Söderfeldt, Peter Lundberg, Maria Engström

**Affiliations:** 1 Department of Clinical and Experimental Medicine, Linköping University, Linköping, Sweden; 2 Center for Medical Image Science and Visualization (CMIV), Linköping University, Linköping, Sweden; 3 Department of Medical and Health Sciences, Linköping University, Linköping, Sweden; 4 The National Board of Forensic Medicine and Linköping University, Linköping, Sweden; 5 Department of Clinical Science and Education, Karolinska Institutet, Stockholm, Sweden; 6 Radiation Physics, Department of Medical and Health Sciences, Linköping University, Linköping, Sweden; 7 Radiology, Department of Medical and Health Sciences, Linköping University, Linköping, Sweden; Hangzhou Normal University, CHINA

## Abstract

Knowledge about the neural underpinnings of the negative blood oxygen level dependent (BOLD) responses in functional magnetic resonance imaging (fMRI) is still limited. We hypothesized that pharmacological GABAergic modulation attenuates BOLD responses, and that blood concentrations of a positive allosteric modulator of GABA correlate inversely with BOLD responses in the cingulate cortex. We investigated whether or not pure task-related negative BOLD responses were co-localized with pharmacologically modulated BOLD responses. Twenty healthy adults received either 5 mg diazepam or placebo in a double blind, randomized design. During fMRI the subjects performed a working memory task. Results showed that BOLD responses in the cingulate cortex were inversely correlated with diazepam blood concentrations; that is, the higher the blood diazepam concentration, the lower the BOLD response. This inverse correlation was most pronounced in the pregenual anterior cingulate cortex and the anterior mid-cingulate cortex. For subjects with diazepam plasma concentration > 0.1 mg/L we observed negative BOLD responses with respect to fixation baseline. There was minor overlap between cingulate regions with task-related negative BOLD responses and regions where the BOLD responses were inversely correlated with diazepam concentration. We interpret that the inverse correlation between the BOLD response and diazepam was caused by GABA-related neural inhibition. Thus, this study supports the hypothesis that GABA attenuates BOLD responses in fMRI. The minimal overlap between task-related negative BOLD responses and responses attenuated by diazepam suggests that these responses might be caused by different mechanisms.

## 1. Introduction

Blood oxygen level dependent (BOLD) responses in functional magnetic resonance imaging (fMRI) reflect hemodynamic responses to neural activation [1, 2], and the responses can become either positive or negative in relation to baseline activity. Positive BOLD responses can be explained by activated neurons that cause increased metabolic demands with accompanying increases of cerebral blood flow and volume [3]. Recent findings, however, suggest that positive BOLD responses are associated with vasodilatation mediated by the excitatory neurotransmitter glutamate [4–6]. However, there are only limited data that explain the neurovascular mechanisms to the frequently observed negative BOLD responses in fMRI [4, 7–9].

A few studies indicate an association between BOLD responses and the inhibitory neurotransmitter gamma-aminobutyric acid (GABA) [8, 10–12]. In these studies, GABA concentrations were measured during rest with magnetic resonance spectroscopy (MRS), and the BOLD responses were measured subsequently during visual or emotional processing. It has also been shown that a positive allosteric modulator of GABA (GABA-PAM) further lowers the negative BOLD response during emotional processing [13]. Other studies have reported dose-dependent relations between GABA-PAMs and decreased cerebral blood flow [14,15], and dose-dependent attenuation of BOLD responses during emotional processing [16]. However, pharmacodynamics vary widely between individuals, and the relationship between the effective plasma concentrations of GABA-PAMs and BOLD responses has not been investigated yet.

In the current study, we have therefore investigated the relation between plasma concentrations of the GABA-PAM diazepam (DZ) and the BOLD responses in healthy subjects during executive processing. In a previous study we investigated the potential effects of DZ on brain activation, and found no drug-related effects [17]. However, in that study we did not examine negative BOLD responses and we did not take individual plasma concentrations of the GABA-PAM into account. Given lead by previous studies reporting correlations between the GABA system and the vascular response in the cingulate cortex [12, 14], the paramount role of the cingulate cortex in executive processing [18], and the relation between GABA levels measured by MRS and negative BOLD responses in the default mode network (DMN) during working memory processing [19], we decided to investigate the effect of the GABA-PAM DZ on BOLD responses in the cingulate cortex during working memory performance. More specifically, we aimed to investigate if pharmacologic GABAergic modulation influences BOLD responses in task-related cingulate regions, or if the pharmacologically modulated responses occur elsewhere in the cingulate cortex. This research contributes importantly to the understanding of the neural mechanisms behind the BOLD response; an issue, which recently has gained much interest and is a matter of debate.

## 2. Materials and Methods

### 2.1 Subjects

Twenty healthy adults (10 males and 10 females) aged between 20 to 30 years (mean age = 25 years) were recruited to the study by advertisement. Two of the female subjects were left-handed; the other 18 subjects were right-handed. In a questionnaire administered before the study process, the subjects signed a declaration of health indicating no health problems that could interfere with the study. The questionnaire included questions about alcohol or drug abuse, cognitive impairments such as memory and attention dysfunction, brain surgery, and present use of pharmaceuticals. All subjects signed informed consent and the study was approved by the Regional Ethical Review Board in Linköping (Dnr 03–528) and the Swedish Medical Products Agency.

### 2.2 Study design

The study had a double blind, randomized, counter-balanced, crossover, and placebo-controlled design. A randomization list and envelopes containing either compressed and non-coated DZ or placebo tablets were prepared by a third party not participating in the study (Apoteket AB, Sweden). Each subject received 5 mg DZ perorally at one fMRI session and placebo at the other. The distribution half-life of DZ is approximately 40 minutes. We used DZ at low dose to obtain ‘minimal sedation’, which has been defined as ‘normal response to verbal stimuli’; *i*.*e*., the subject responds normally to verbal commands although cognitive functions may be impaired (American Society of Anesthesiologists, ASA). As the study had a counter-balanced design, half of the subjects received DZ on the first fMRI session and the other half on the second session.

### 2.3 Procedure

All subjects went through three separate MRI examinations on three different occasions, namely: 1) structural MRI, 2) DZ fMRI, and 3) placebo fMRI. On the first occasion, the subjects were informed about the study, and they were examined by structural MRI in order to allow them to be acquainted with the MRI environment. Both fMRI sessions were separated by at least one, and at most six weeks. During the fMRI sessions, three different tasks in randomized order for each subject were administered: 1) working memory (WM), 2) word fluency, and 3) finger tapping (results of the word fluency and finger tapping tasks are not reported here).

The subjects were instructed not to take alcohol or other drugs for 24 h before the fMRI examinations. In addition, they were firmly instructed not to intake products containing caffeine or nicotine on the examination day. At the fMRI session, the subjects were administered DZ or a placebo approximately 30 minutes before the scanning started. Blood plasma samples for measurements of DZ concentration were collected immediately before and approximately 45 minutes (range 37–68 minutes) after the administration of the drug. We aimed to collect blood samples in the middle of the investigation in order to obtain diazepam concentrations as close to the fMRI task as possible. However, due technical issues and previous scans not being completed in time the second blood samples were sometimes drawn later than planned.

### 2.4 Blood sample analysis

Plasma samples were analyzed for DZ concentration at the National Board of Forensic Medicine, Linköping, Sweden. After adding prazepam as an internal standard, each sample was extracted at pH 7. The extract was analyzed with gas chromatography with a nitrogen-phosphorus detector (Hewlett Packard 5890 Series II GC, Hewlett-Packard Company, Palo Alto, USA). The chromatograph was temperature-programmed. The accuracy of the analysis method was controlled by repeated measurements: five replicates of two low-concentration samples (0.005 and 0.010 mg/g). The coefficient of variation (CV) was 4.08% and 2.00% respectively. More details of the analytic method are reported elsewhere [20, 21].

### 2.5 Task design

The n-back working memory paradigm had a block design with 30 s in each block. The paradigm consisted of three blocks: 0-back, 1-back, and 2-back. All blocks were presented in randomized order with four repetitions of each block. Periods with fixation on a crosshair during 20 s were administrated before the first task set, comprising three n-back blocks presented in randomized order, and after each task set. During the n-back tasks, series of letters were displayed one at a time and the subjects were asked to determine: a) if the current letter was the same as a specified target letter (0-back or baseline); b) if the current letter was the same as the previously presented letter (1-back); c) If the current letter was the same as the letter presented two letters previously (2-back). Each letter was presented for 0.5 s followed by a blank screen, which was displayed for 1.5 s. Each n-back block comprised 15 events (= presented letters), of which four were targets. The level of the upcoming n-back task was indicated on the screen during 5 s before each task block. Total duration of the task was 8 min and 40 s.

The tasks were presented using a back projection screen viewed via a mirror mounted on the head coil. The participants responded to the given task using a response box (Photon Control Inc., Burnaby, Canada). Response time and accuracy were measured as behavioral performance. The fMRI paradigm was designed using the SuperLab software (Cedrus Corporation, San Pedro, USA).

### 2.6 fMRI data acquisition

An echo planar imaging (EPI) gradient echo sequence optimized for BOLD contrast was used to acquire fMRI data on a Philips Achieva 1.5 T scanner (Philips Healthcare, Best, The Netherlands). The following image parameters were used: Data matrix = 80x80, Field of View (FOV) = 230 mm, Echo Time (TE) = 40 ms, Repetition Time (TR) = 2700 ms, Flip angle = 90°, Number of slices = 31, Slice thickness = 3 mm, Number of volumes = 193. Axial slices aligned between the floor of sella turcica and the corner of the fourth ventricle were acquired in interleaved order.

### 2.7 fMRI data analysis

#### 2.7.1 Preprocessing

Pre-processing and first level analysis of the fMRI data were performed using SPM2 (Wellcome Department of Imaging Neuroscience, University College, London, UK), employing the same method as described in a previous paper by us [17]. The images were firstly corrected for different acquisition timing using sinc interpolation. Thereafter the images were realigned to the mean image to correct for subject movement during scanning. The images were also corrected for movement-related susceptibility effects using generative models as described by Andersson and co-workers [22]. Finally, the images were normalized to Montreal Neurological Institute (MNI) standard space with voxel size 3x3x3 mm^3^, and smoothed with an 8 mm Gaussian filter to ameliorate differences in intersubject localization.

#### 2.7.2 First level analysis

The EPI images were analyzed using a general linear model (GLM) implemented in SPM8. To estimate the BOLD responses in each subject, we used the canonical hemodynamic response function implemented in SPM8 and its temporal derivative. Each condition of the n-back task (0-back, 1-back, 2-back) was modeled separately in the GLM analysis. The fixation period was regarded as a short pause and thus not modeled explicitly. Parameters from the movement correction were included as nuisance variables in the GLM to statistically control for signal change related to head motion. In addition, we applied a high-pass filter with cut-off at 128 s and the AR(1) autocorrelation model type. Here, we estimated the BOLD responses during the most difficult condition of the WM task. We did so by calculating contrast images representing differences in the BOLD responses between the 2-back condition and the 0-back condition, which was regarded as a controlled low-level activation baseline. The contrasts were based on the hemodynamic response function and not on its temporal derivative, which was included as a nuisance variable.

#### 2.7.3 Second level analysis

In the second level analysis in SPM8, we assessed whole brain positive and negative BOLD responses by one-sample t-tests of random effects using the contrast images from the placebo session. Group-related differences in brain responses between the DZ and the placebo sessions were obtained from paired t-tests of the contrast images. In this way we could analyze for regions that were more activated during the DZ session compared to the placebo session and vice versa.

We aimed to investigate which regions of the brain that had BOLD responses that were directly correlated to DZ concentration. Therefore, the plasma DZ levels were included as covariates of interest in the second level analyses of the DZ session. In this way, the correlations between BOLD responses and DZ concentrations in each image voxel were calculated. The contrast estimates from the image voxel with highest correlation between BOLD responses and DZ concentrations were extracted for each subject as measures of brain responses to DZ treatment for pharmacokinetic/pharmacodynamic (PK/PD) modeling (see section 2.12).

### 2.8 Regions of interest

In this study, we aimed to investigate if DZ attenuates brain activation in the cingulate cortex during a working memory task. Therefore we did small volume corrections within the entire cingulate cortex to investigate if there were any pure task-related effects or evidence for DZ-modulation of brain activation during the working memory task. In follow-up analyses, we investigated these effects in cingulate subregions by making statistical analyses in predefined regions of interest (ROI) in the cingulate cortex based on cytoarchitecture and functional studies. The cingulate cortex is divided into four main sub-regions with different cytoarchitectures and functions: the anterior cingulate cortex (ACC), the midcingulate cortex (MCC), the posterior cingulate cortex (PPC), and the retrosplenial cortex [23]. The ACC is further subdivided into a subgenual (sACC) and a pregenual (pACC) portion, which are thought to be involved with affective and autonomic responses, and emotional integration, respectively [18, 23]. The MCC is also subdivided into two different compartments: the anterior MCC (aMCC), which is involved in cognition, and the posterior MCC (pMCC), which is involved in skeletomotor orientation [24]. The role of the PCC is multifaceted; it is thought to be involved in diverse functions such as autobiographical memory, spatial learning, and mind wandering [25]. Image masks of the PCC (size = 122 voxels), pMCC (size = 515 voxels), aMCC (size = 217 voxels), pACC (size = 180 voxels), and the sACC (size = 68 voxels) were constructed from the Wake Forrest University (WFU) Pick Atlas [26] and the MarsBaR ROI toolbox [27] based on the descriptions in [23]. Voxels sizes are given as mean value of left and right hemisphere ROIs, since the ROIs were slightly asymmetric.

### 2.9 Significance Thresholds

At the whole brain level of analysis of task-related effects during the placebo session, the resulting images were preliminarily thresholded at uncorrected p = 0.001. In the ROI analyses, we used a somewhat lower preliminary threshold, p = 0.01. In the full-scale significance estimation of all analyses, we firstly investigated if there were any significant effects in the entire cingulate cortex cluster using the criterion p < 0.05, family-wise error (FWE) corrected for multiple comparisons. Results from the follow-up analyses in cingulate subregions are reported as significant if peak activation p < 0.05 FWE corrected in each ROI and as trends if p < 0.1 FWE corrected.

### 2.10 Movement analysis

Since motion is known to influence fMRI data we made an estimation of the average motion during the working memory scan by calculating absolute value means of the six movement parameters (x, y, and z displacements and pitch, role, and yaw rotations) obtained from the movement correction of the fMRI images. Rotational parameters are given in radians (ø) and they were converted to millimeters by calculating the displacement on a circle (*s*) with radius, *r* = 50 mm, *s* = *rø*. The radius, r, is an approximation of the average distance from the cerebral cortex to the center of the head [28].

### 2.11 BOLD response curves

In order to estimate if DZ-modulated responses were positive or negative with respect to baseline, we extracted the BOLD responses in the aMCC from normalized images of three participants with DZ plasma concentration > 0.1 mg/L, using a spherical mask with radius 5 mm centered at [0, 18, 21]. Linear trends in the BOLD time series were removed using the detrend command in Matlab R2015a (MathWorks, Natick, MA, USA). BOLD responses from all tasks (0-back, 1-back, and 2-back) where extracted for those task sets where the 2-back task was administered immediately after the fixation period; allowing normalization of the BOLD response curves by subtracting the signal value at the last time point of the fixation period from all data points of the task set time series. The same procedure was performed for both the placebo and the DZ sessions.

### 2.12 Pharmacological Analysis

#### 2.12.1 Data normalization

The plasma DZ concentrations (measured in mg/L) were normalized to body weight (mg/kgBW). Normalization was performed with Nadler’s formula for blood volume, which is based on weight, height, and sex [29], and haematocrit levels of 46% and 42% for males and females respectively were assumed. Furthermore, the BOLD response data was rescaled to run from 0 to 1, where ‘0’ was defined as 125% of the maximal response below zero and ‘1’ was defined as 125% of the maximal response above zero for all subjects.

#### 2.12.2 Pharmacological model based analysis

A PK/PD model was used for the model based pharmacological analysis. The purpose was two-fold: (i) to control for variable time between blood sampling and fMRI-measurement, and (ii) to model the physiological effect of DZ on neural activation.

1. In the PK/PD model the elimination of DZ, during the time between the venous blood sampling and the fMRI acquisition, was described by a mono-exponential function:
  $$C_{P}=C_{P}^{0}e^{-k_{el}\Delta \tau }$$ (Eq. 1)
  where *C*_*P*_ is the estimated DZ plasma concentration at the fMRI WM experiment, *C*_*P*_^*0*^ is the DZ plasma concentration as measured in the venous blood sample approximately 45 min after DZ administration, *k*_*el*_ is the elimination constant, and Δτ is the time difference between venous blood sample and the fMRI WM experiment. The elimination constant was found to be negligible in an initial model parametrization. As a result the elimination of DZ during the time between the venous blood sampling and the fMRI acquisition was in a practical sense non-existing. Therefore the plasma concentration of DZ at the time of the fMRI acquisition was assumed to be equal to the DZ concentration measured in the venous blood.
2. The physiological effect of DZ was in the PK/PD model described by a Hill equation, which assumes that the drug effects are directly proportional to receptor occupancy as well as the assumption that the plasma drug concentrations are in rapid equilibration at the receptor site [30]:
  $$Effect=E_{0}-\frac{E_{\text{max}}\;*C_{P}}{EC_{50}+C_{P}}$$ (Eq. 2)
  where, due to the normalization of the data, the base line effect (*E*_*0*_) was set to 1, the maximal effect (*E*_*max*_) was also set to 1, whereas the drug concentration yielding the half maximal effect (*EC*_*50*_) was an free parameter in the model parameterization.

The PK/PD model was then parameterized using a nonlinear global optimization method (Differential Evolution [31]). The PK/PD model based analysis was performed using Mathematica (10.0.2.0, Wolfram Research Inc., Champaign, IL, U.S.).

## 3 Results

### 3.1 DZ did not affect behavior

There were no significant differences in reaction time or performance between the placebo and DZ sessions. The mean reaction times during the 2-back task were 613 ms and 595 ms for the placebo and DZ session, respectively. The accuracy was 98.2% during the placebo session and 98.5% during the DZ session. For more detailed information on behavioral results see Ragnehed and co-workers [17]. Since DZ might influence the participants’ motion during scanning we estimated the average motion parameters in both placebo and DZ sessions. There was no significant difference in average motion between DZ and placebo runs (p = 0.084), although it seemed like the participants moved less during the DZ session (0.12 mm in average) compared to the placebo session (0.15 mm). It should, however, be noted that average motion was submillimeter in this experiment and no participant moved more than a voxel size. Importantly for our work, there were no significant correlations between DZ concentration and average motion (p = 0.633).

### 3.2 DZ plasma concentration varied in individual subjects

In this study, we investigated the effective DZ plasma concentration in each subject before DZ or placebo intake and after approximately 45 minutes during a pause in the fMRI scanning. At the zero point checking of DZ concentration before the first fMRI session, none of the subjects had a measurable DZ plasma concentration. All subjects were given the same dose of DZ (5 mg). Nevertheless, we observed a (desired) variation in DZ plasma concentration among the subjects, ranging from zero to 0.16 mg/L. The observed time course and plasma concentrations agreed with what has been reported previously [32]. Self-rated sedative effects reached an optimum around 0.5–2 h after DZ-intake, which also agreed with the experimental design described here.

### 3.3 WM whole brain analyses

#### 3.3.1 WM elicited positive BOLD responses in the executive network

**Fig 1:**
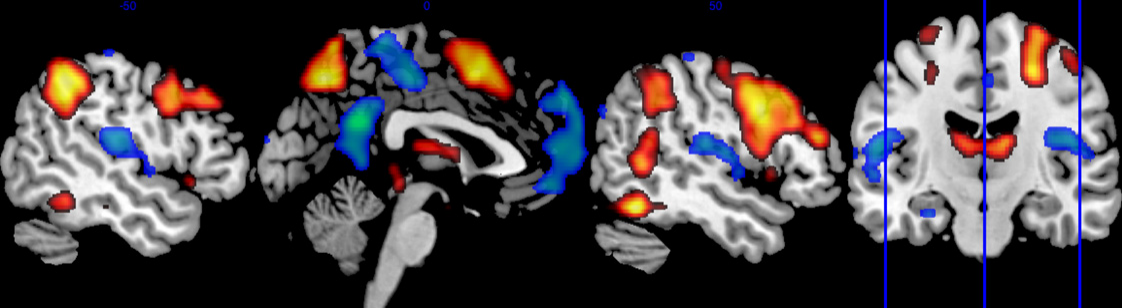
Positive and negative BOLD responses during executive processing. The sagittal brain images show whole brain group activation during the working memory task. Areas in orange/red denote positive BOLD responses, which were mainly found in the executive network. Areas in green/blue denote negative BOLD responses, which were mainly found in the default mode network. The coronal image shows the position of visualized sagittal slices.

As expected from previous studies [33], the WM task elicited brain activation in the executive network (Fig 1). Data from whole-brain analysis showed significant (p < 0.05 FWE corrected for multiple comparisons) task-related positive BOLD responses in several areas of the executive network: the bilateral posterior parietal cortex, dorsolateral prefrontal cortex, and the medial frontal cortex including the supplementary motor area (SMA) and adjacent aMCC. Task-related positive BOLD responses were also found in the bilateral inferior temporal lobe and the thalamus.

#### 3.3.2 WM elicited negative BOLD responses in the default mode network

We made a whole-brain analysis for task-related responses that were negative with respect to the baseline (0-back). Data showed significant (p < 0.05 FWE corrected for multiple comparisons) clusters with negative BOLD responses in cortical midline structures corresponding to the DMN: most prominently in the PCC and the medial frontal cortex, see Fig 1. Clusters with negative BOLD responses were also found in bilateral temporo-parietal areas.

### 3.4 WM elicited negative BOLD responses the cingulate cortex

**Fig 2:**
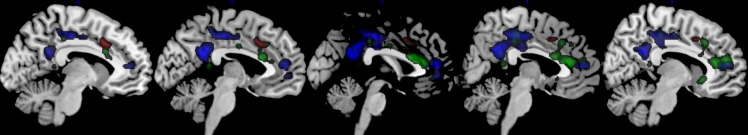
Task- and DZ-related BOLD responses in the cingulate cortex. The sagittal brain images show regions with task-related positive (red) and negative (blue) BOLD responses in the cingulate cortex in the whole group during the working memory task. Regions where the BOLD responses correlated inversely to diazepam plasma concentration are visualized in green. Task-negative and diazepam-attenuated responses were significant after family wise error (FWE) correction for multiple comparisons. Task-positive responses in the cingulate cortex were not significant after FWE correction.

Data showed significant task-related negative BOLD responses in the cingulate cortex, p < 0.001, FWE corrected. We found negative responses in following sub-regions: the bilateral pACC corresponding to BA 32 (MNI co-ordinates = [0, 51, 9], p = 0.002), the bilateral PCC in BA 31 (MNI co-ordinates = [0, –51, 27], p < 0.001), and the bilateral pMCC, BA 31 (MNI co-ordinates = [3, –21, 48], p = 0.006); see Fig 2. All ROI analyses were based on preliminary uncorrected statistical threshold of p = 0.01 and FWE small volume correction in each ROI. We found no cingulate clusters of significant task-positive responses after correction for multiple comparisons, p = 0.27.

### 3.5 DZ lowers BOLD responses in the cingulate cortex

**Fig 3:**
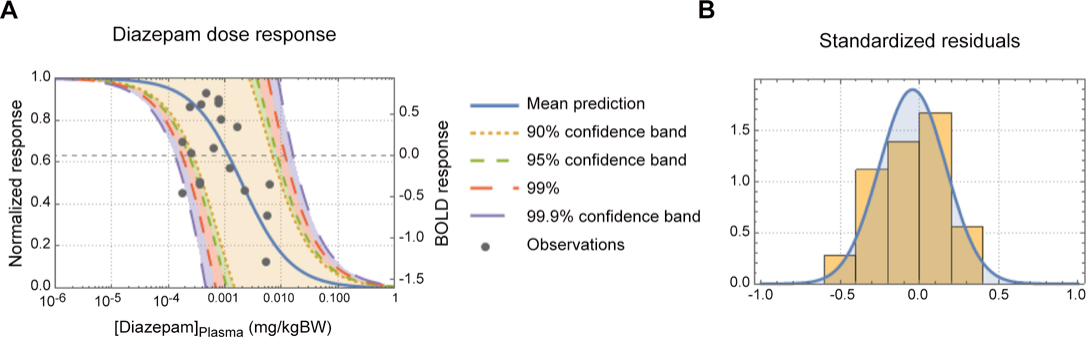
DZ dose-response curve. (A) Panel A shows the predicted diazepam-dose-fMRI response curve. The normalized response is shown on the left y-axis and the actual BOLD response on the right y-axis. The mean prediction and a range of prediction confidence bands are also shown in panel A. (B) Panel B shows the standardized residual of the model fit.

The modelled plasma DZ *vs*. WM brain responses dose response curve is shown in Fig 3. As can be seen in Fig 3A, the dynamic dose range was about 0.1 to 10 mg/kgBW (body weight). The conventional PK/PD model was able to describe about 90% of the variance in the data (R^2^ = 0.902) and as can be seen in Fig 3B the residuals followed an expected normal distribution.

A small volume correction of the entire cingulate region showed that we had significant inverse correlation between BOLD responses and individual DZ plasma concentrations, cluster p = 0.006, FWE corrected. A follow-up ROI analysis in sub-regions of the cingulate cortex showed that DZ modulated the BOLD responses in the bilateral aMCC (medial BA 24) and in the right pACC (BA 32 and medial BA 24), see Table 1, Fig 2. We also found trends (p < 0.1, FWE corrected) of inverse correlations between the BOLD responses and DZ concentrations in the bilateral PCC, and the right sACC and pMCC.

**Table 1:** Inverse correlation between DZ concentration and BOLD response in the cingulate cortex. The table shows statistics and the location of the peaks in sub-regions of the cingulate cortex where the BOLD response was inversely correlated with DZ plasma concentrations. L = left, R = right. The cluster size denotes the number of inversely correlated voxels in the region of interest (ROI). The p-value is the family-wise error (FWE) corrected p-value in the correlation peak. The co-ordinates (x, y, and z) are given in the Montreal Neurological Institute (MNI) space. All ROI analyses were based on preliminary uncorrected statistical threshold of p = 0.01 and FWE small volume correction in each ROI.

ROIs^1^		Cluster Size	Peak *p* (FWE)	*x*	*y*	*z*
sACC	R	14	0.069	9	21	-6
pACC	R	79	**0.044**	12	45	15
aMCC	L	38	**0.016**	0	18	21
aMCC	R	31	**0.030**	3	15	21
pMCC	R	22	0.069	3	-30	39
PCC	L	6	0.057	-6	-45	9
PCC	R	20	0.064	6	-48	27

^**1**^ sACC = subgenual Anterior Cingulate Cortex, pACC = pregenual Anterior Cingulate Cortex, aMCC = anterior MidCingulate Cortex, pMCC = posterior MidCingulate Cortex, PCC = Posterior Cingulate Cortex.

**Fig 4:**
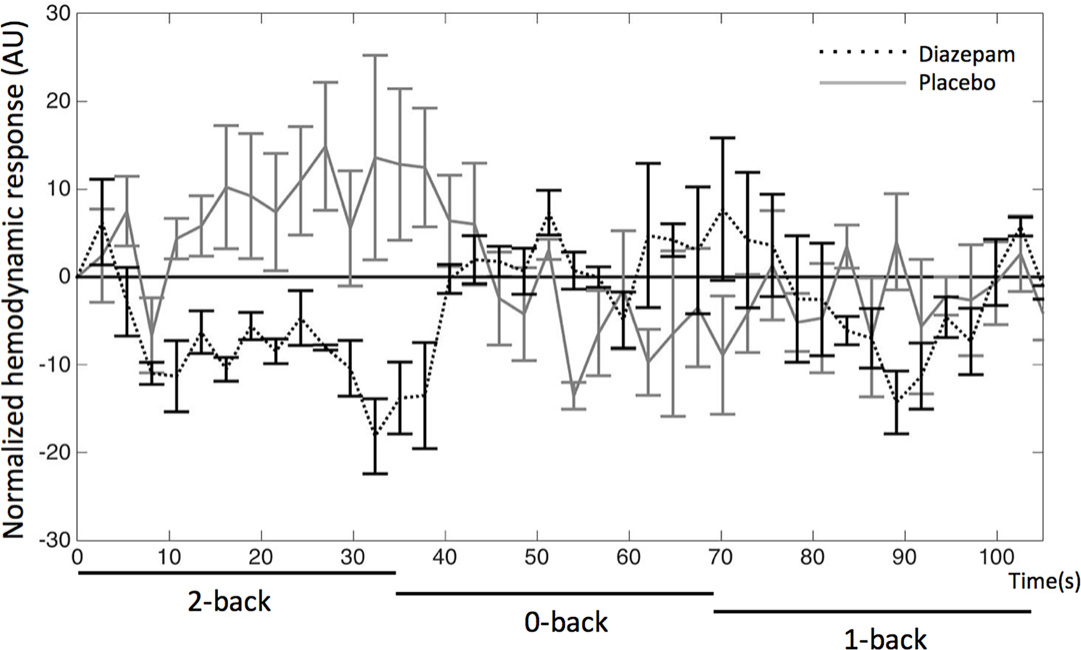
DZ-related negative BOLD responses. The figure shows mean BOLD responses in the anterior midcingulate cortex [0, 18, 21] for three subjects with DZ plasma concentration > 0.1 mg/L during the 2-back, 1-back, and 0-back tasks. The BOLD responses are normalized to baseline defined as the fixation period immediately before the 2-back task. The figure shows that the BOLD responses are negative with respect to baseline during the DZ session and positive during the placebo session.

Fig 3A shows the inverse dose-response correlation between the BOLD response in the aMCC and DZ concentration. From the dose-response curve it can be seen that half maximal effect (*EC*_*50*_) of DZ is 2.0 μg/kgBW and a tenth of the maximal effect (*EC*_*10*_) is 0.2 μg/kgBW. Two subjects were removed from the dose-response analysis as they did not have any detectable DZ concentration at the time of sampling. Three subjects had plasma DZ concentration > 0.1 mg/L and all of them had aMCC negative BOLD responses in comparison to baseline activity defined as the fixation period administered immediately before the 2-back task condition (Fig 4). In Fig 4, it is also shown that BOLD responses to the 2-back task administered during the placebo session are positive with respect to the fixation baseline. None of the pre-defined ROIs showed activation that was positively correlated to individual DZ plasma concentration.

There were no significant differences between the DZ and the placebo sessions in terms of BOLD responses in the cingulate cortex (PCC, MCC, or ACC) when comparing the two sessions without taking the individual DZ concentrations into account.

### 3.6 Pure task-negative and DZ-attenuated BOLD responses did sparsely overlap

We observed only a sparse overlap between regions with pure task-related BOLD responses and regions where the BOLD responses were modulated by DZ. In Fig 2, it is seen that the task-related negative BOLD responses were mainly found in anterior and posterior portions of the cingulate cortex. The BOLD responses attenuated by DZ appeared primarily in cingulate regions adjacent to the corpus callosum with their main foci separated from negative task-related responses. However, we observed sparse overlap between DZ-modulated responses and pure negative BOLD responses, primarily in the left pACC, but also in small PCC clusters.

## 4 Discussion

We evaluated the modulatory effect of low doses of the GABA-PAM DZ on the BOLD responses in cingulate cortex sub-regions. The main findings were (1) DZ attenuated BOLD responses in the cingulate cortex; (2) DZ-modulated responses were task-dependent; and (3) Regions with DZ-modulated responses did for the most part not overlap with cingulate regions with pure task-related BOLD responses.

### 4.1 GABA-PAMs and BOLD responses

In the present study, we found an inverse sigmoidal dose-response correlation between DZ concentrations and BOLD responses in the cingulate cortex. Hence, we showed that the GABA-PAM DZ clearly attenuates the BOLD responses under these conditions. Moreover, in subjects with high DZ concentration, the BOLD responses were negative in comparison to baseline activity (Fig 4). We interpret these results being caused by GABA-related neural inhibition.

This conclusion is supported by previous studies showing that benzodiazepines reduce BOLD responses in the orbitofrontal cortex [13] and the amygdala [16] during emotional processing. Previous studies also show that DZ causes BOLD reduction in the midcingulate cortex during a reaction time task [34].

### 4.2 GABA-related neural inhibition and BOLD responses

We observed an inverse sigmoidal dose-response correlation between DZ concentrations and BOLD responses in the cingulate cortex. Furthermore, subjects with high DZ concentration had negative BOLD responses with respect to the fixation baseline (Fig 4). Prior studies have shown a general GABA-related reduction of cerebral blood flow [14, 15]. Reduction of cerebral blood flow is typically connected to increased BOLD responses [3]. However, if the blood flow reduction is caused by neural inhibition, this will lead to attenuated or negative BOLD responses [35]. Therefore, we interpret our findings being caused by GABA mediated neural inhibition (see also discussion in Section 4.4).

GABA is the main inhibitory neurotransmitter of the brain and is therefore essential in the regulation of synaptic activity. GABA-PAMs enhance neural inhibition by increasing chloride influx through the GABA_A_ receptor complex [36]. However, the relation between inhibitory neurotransmitters and the BOLD response is a matter of debate.

The BOLD response depends on changes in local deoxyhemoglobin content, which are caused by two separate effects: 1) the hemodynamic response related to increased cerebral blood flow as well as volume, 2) the metabolic response related to increased cerebral metabolic rate of oxygen (CMRO_2_) [37]. The current view is that a positive BOLD response is related to synaptic activity mediated by glutamate in excitatory neurons [4]; however, it has been shown that inhibitory neurons also produce increased BOLD responses in some cases [8]. Although negative BOLD responses have been explained by decreases in cerebral blood flow due to blood flow redistribution [38] or increases in CMRO_2_ without accompanying cerebral blood flow increase [39], previous research suggest that negative BOLD responses are related to neural inhibition [9, 35].

Human and animal studies suggest that negative BOLD responses are caused by direct neural inhibition. Elevated endogenous GABA levels have been shown to correlate with decreased BOLD responses in rats [40], indicating that negative BOLD responses are associated with GABA-related neuronal inhibition. In humans, Northoff and co-workers demonstrated that the resting-state GABA concentrations in ACC predicted the amount of negative BOLD responses in subsequent fMRI; the higher total concentration of GABA, the stronger the negative BOLD response became, suggesting that GABA mediates negative BOLD responses in the ACC [12]. A recent study by Hu and co-workers also reported that endogenous GABA levels correlate inversely with BOLD responses in the DMN during a working memory task [19].

### 4.3 GABA-related BOLD responses in the cingulate cortex

A striking feature of our results is the sparse overlap between sub-regions with pure *task-related* BOLD responses and sub-regions with *DZ-modulated* responses. That is to say, the clusters in pACC and aMCC where DZ attenuated the BOLD responses were not primarily found in sub-regions showing pure task-related negative BOLD responses. Therefore, this suggests that the structure and location of the DZ receptor might differ from the GABA receptor that is responsible for the pure task-related negative BOLD response in the cingulate cortex. This might be in part due to the known variation of GABA receptor concentration across the human brain [41] and across the cingulate cortex [42, 43].

We observed significant task-related negative BOLD responses in pACC and in the posterior parts of the cingulate cortex (pMCC and PPC). The pACC is a cingulate cortical area with a higher concentration than average of GABA_B_ receptors [42, 43]. Receptor architecture studies have also shown that pMCC and PPC have higher than average density of both GABA_B_ and non-benzodiazepine binding GABA_A_ receptors [42, 43]. In our work, areas with pure task-related negative BOLD responses were heterogeneously distributed across the cingulate cortex and partly coincided with cingulate areas with high density of GABA receptors.

### 4.4 GABA and cerebral blood flow

It is well known that GABA-PAMs influence cerebral blood flow [44] and prior imaging studies have found decreased cerebral blood flow in relation to GABA-PAMs [14, 15] and endogenous GABA levels measured by MRS [45, 46], but see also [47]. Relevant to our study, GABA-PAM mediated reduction of cerebral blood flow has specifically been observed in the cingulate cortex [14, 48, 49]. Krause and co-workers also reported strong inverse correlation between GABA levels measured in the ACC and whole-brain blood flow [45] indicating that our results might be caused by vascular effects of DZ unrelated to neural activity. As regions where we found significant inverse correlations between DZ and BOLD responses are located in brain areas with large vessels, we made post-hoc analyses of BOLD responses in the anterior insula where the middle cerebral artery passes through. In the anterior insula, we found no significant DZ-effects or trends thereof. We also made post-hoc analyses of DZ-related responses in the cingulate and anterior insular cortices during the 1-back condition in our working memory task, and also during the verbal fluency task administered in this study (descriptions of the verbal fluency task is found in [17]). We found no trends of DZ-modulated responses in either of these post-hoc analyses. We therefore conclude that DZ modulation of the BOLD response is neural in origin and related to the task performed during fMRI.

### 4.5 Negative BOLD response and the default mode network

In the present study we found task-related negative BOLD responses in pACC, pMCC, and PCC during executive processing. Anterior and posterior regions of the cingulate cortex are considered to be parts of DMN as suggested by Raichle and co-workers [50]. DMN is defined as the brain network that is most active during the ‘resting state’, when the individual is mind wandering and not focusing on external stimuli [51]. When an individual is focusing on external stimuli, the regions belonging to the DMN consistently show negative BOLD responses [52], or down-regulation of these regions. These task-related negative BOLD responses are believed to optimize performance by reducing interference from task-irrelevant areas in the brain [53, 54]. It has also been demonstrated that the more difficult the task is, the greater the negative BOLD response becomes [55, 56]. Moreover, momentary lapses in attention during cognitive tasks have been associated with less negative BOLD response in the DMN [57]. Greicius *et al*. studied DMN connectivity during conscious sedation with a GABA-PAM and found that DMN functional connectivity persisted during sedation [58].

### 4.6 Strengths and limitations

The present study had several methodological strengths. The risk of an intersession effect was reduced by an initial adaptation scan, and the number of subjects was adequate (n = 20). Furthermore, the administered DZ dose was low, ensuring minimal sedation and unperturbed normal response to verbal stimuli. The small modulatory doses of DZ given in the present study did not significantly influence the subjects’ performance, measured as the number of correct responses and reaction time. This was in our view an important aspect of the experimental design. Nevertheless, it is known that benzodiazepines influence performance in a variety of tasks [34]. Especially, it is known that benzodiazepines impair higher cognitive function such as attention, memory, or planning [59–61]. One limitation of the present study was that we did not investigate the subjects’ cognitive function by also administrating more challenging tasks, which might have shown DZ-related performance decreases.

Another particular strength of our study is that DZ plasma concentration was modeled using a non-linear dose-response analysis, to retrospectively control for individual variations in DZ metabolism. However, although small, non-sedative doses of DZ are often prescribed as calming drugs, the low DZ plasma concentration in some subjects, may nevertheless suggest that more individualized dosage by for example controlling for body weight, length and sex could be explored in future studies. The pharmacokinetic model in the present study suggests that DZ plasma concentration of at least 2.0 μg/kgBW is necessary to obtain 50% of the maximal BOLD response. Although we did control for individual variability regarding pharmacokinetics at the actual time of the experiment, it did not affect the results. According to a pharmacokinetic study by Friedman and co-workers the peak DZ plasma concentration occurs after 0.5 h after administration [62]. In that study it was also shown that the plasma concentration increases rapidly to a maximum and thereafter decays slowly during several few hours. Shader *et al*. reported similar observations [32]. It is possible that the blood samples for DZ analysis were taken too early after administration in some subjects. It is also possible that some subjects had a full stomach, which might have slowed the absorption. Another limitation of the present study was that the subjective levels of consciousness were not explicitly measured (such as by self-rating of the sedative effect), although reaction time and task performance were determined.

## 5 Conclusions

In summary, the present study demonstrated inverse dose-response correlation between DZ concentration and BOLD response in the cingulate cortex. Our interpretation is that the inverse correlation between DZ and the BOLD response was caused by GABA-related neural inhibition. A particularly interesting finding was that brain regions with DZ-related BOLD responses did only sparsely overlap with brain regions demonstrating task-related negative BOLD responses. The minor overlap between task-related BOLD responses and responses attenuated by DZ suggests that these responses might be caused by different mechanisms.

## Supporting Information

S1 Dataset The supporting information file PlosOneDataDZ.zip contains image and header files relevant to the current manuscript. The file 2-backPositive.img contains results from the one-sample t-test of brain activation during the 2-back working memory task obtained during the placebo session. The file 2-backNegative.img contains results from the one-sample t-test of the negative blood oxygen level (BOLD) responses during the working memory task also obtained during the placebo session. The file 2-backDZcorrelationInverse.img contains the results from the inverse correlation between working memory activation and diazepam plasma concentration. Finally, the files WordDZcorrelationInverse.img contains results from the inverse correlation between brain activation during the word generation task and diazepam plasma concentration discussed in Section 4.4.
(ZIP)
